# Heightened Vulnerability to MDR-TB Epidemics after Controlling Drug-Susceptible TB

**DOI:** 10.1371/journal.pone.0012843

**Published:** 2010-09-22

**Authors:** Jason D. Bishai, William R. Bishai, David M. Bishai

**Affiliations:** 1 School of Humanities and Sciences, Stanford University, Stanford, California, United States of America; 2 Division of Infectious Diseases, Department of Medicine, Johns Hopkins School of Medicine, Baltimore, Maryland, United States of America; 3 Johns Hopkins Bloomberg School of Public Health, Baltimore, Maryland, United States of America; Medical Research Council (MRC) Laboratories, Gambia

## Abstract

**Background:**

Prior infection with one strain TB has been linked with diminished likelihood of re-infection by a new strain. This paper attempts to determine the role of declining prevalence of drug-susceptible TB in enabling future epidemics of MDR-TB.

**Methods:**

A computer simulation of MDR-TB epidemics was developed using an agent-based model platform programmed in NetLogo (See http://mdr.tbtools.org/). Eighty-one scenarios were created, varying levels of treatment quality, diagnostic accuracy, microbial fitness cost, and the degree of immunogenicity elicited by drug-susceptible TB. Outcome measures were the number of independent MDR-TB cases per trial and the proportion of trials resulting in MDR-TB epidemics for a 500 year period after drug therapy for TB is introduced.

**Results:**

MDR-TB epidemics propagated more extensively after TB prevalence had fallen. At a case detection rate of 75%, improving therapeutic compliance from 50% to 75% can reduce the probability of an epidemic from 45% to 15%. Paradoxically, improving the case-detection rate from 50% to 75% when compliance with DOT is constant at 75% increases the probability of MDR-TB epidemics from 3% to 45%.

**Conclusions:**

The ability of MDR-TB to spread depends on the prevalence of drug-susceptible TB. Immunologic protection conferred by exposure to drug-susceptible TB can be a crucial factor that prevents MDR-TB epidemics when TB treatment is poor. Any single population that successfully reduces its burden of drug-susceptible TB will have reduced herd immunity to externally or internally introduced strains of MDR-TB and can experience heightened vulnerability to an epidemic. Since countries with good TB control may be more vulnerable, their self interest dictates greater promotion of case detection and DOTS implementation in countries with poor control to control their risk of MDR-TB.

## Introduction

Multidrug resistant (MDR) tuberculosis affects between 0.5 to 2 million annually [1], [2]. Yet there were only 111 MDR-TB cases reported in the U.S. in 2006 [1] with 82% of U.S. cases foreign born. Both MDR and overall TB caseloads in the U.S. have declined since the 1990s [3], [4]. Although the current low burden of MDR-TB in the US may fuel complacency from speculation that drug resistant mycobacteria lack sufficient virulence to sustain large epidemics, growing caseloads in central Asia suggest otherwise. Ten of the regions with the highest and most rapidly growing prevalence of MDR-TB are all in the former Soviet Union [1]. It is significant that current MDR-TB epidemics in central Asia followed on the heels of modest success in the control of TB in the Soviet Union [5], [6].

Because primary infection with drug susceptible TB confers partial immunity against exogenous re-infection with a different strain, a population may become more vulnerable to the spread of MDR-TB when the prevalence of latent TB infection has been lowered [7]. We hypothesize that improving a population's TB prevalence can have dual effects on the probability of an MDR-TB epidemic. Drug susceptible TB is the substrate from which MDR TB emerges, but it also may confer partial protection against exogenous re-infection with MDR-TB. Our findings suggest that reductions in TB caseloads need to occur rapidly to eliminate the substrate from which MDR strains emerge before lack of immunity is sufficiently widespread to ease the spread of mutant strains. Improving compliance with treatment programs is one of the chief public health tools to lower the prevalence of drug susceptible TB. Under certain circumstances, when treatment compliance is poor, increasing the availability of programs could actually increase the probability of MDR-TB. This paper identifies the features of TB control programs that alter the likelihood of novel MDR-TB strain emergence and the extent of spread of newly emerged resistant strains. In so doing, the paper highlights strategies that achieve the goal of controlling drug susceptible TB while maintaining minimal risk of MDR-TB outbreaks.

## Methods

### Study design

Some tuberculosis simulation models are constructed specifically to forecast disease burdens in the future. For forecasting, validating model output with available empirical observations on recent disease trends is paramount [8], [9], [10]. Other simulation exercises are designed to enhance understanding of the basic biology of the disease [11]. To understand TB biology, one alters biological and environmental parameters to see how much these parameters matter regardless of whether this replicates field data. This study is of the latter type, designed to understand how MDR-TB spread reacts to biological and policy parameters along a continuum that cannot be observed in real world epidemiological data.

The model begins with a stylized population just launching its use of antimicrobials and follows their subsequent 500 years of experience with TB. One could consider the starting conditions to represent an Alaskan or Native American population in the year 1950 prior to widespread treatment with antimicrobials [12]. The simulated population will not experience any migration, there is no age structure, no population growth, no gender, and no co-infections with conditions that alter susceptibility to TB. Also BCG is assumed to either not be in use or to have no protective efficacy. These omitted factors have all had important effects on historical trends in TB, but because they have little direct effect on the emergence of resistant TB their inclusion would raise complexity without helping to address the question of interest. This strategic decision makes it impossible to seek model validation by comparing TB trends in the model to historical trends that were influenced by outside economic factors. Validation depends on selecting model parameters consistent with the known epidemiology of TB transmission and ensuring that the model responds appropriately to these parameters.

**Figure 1:**
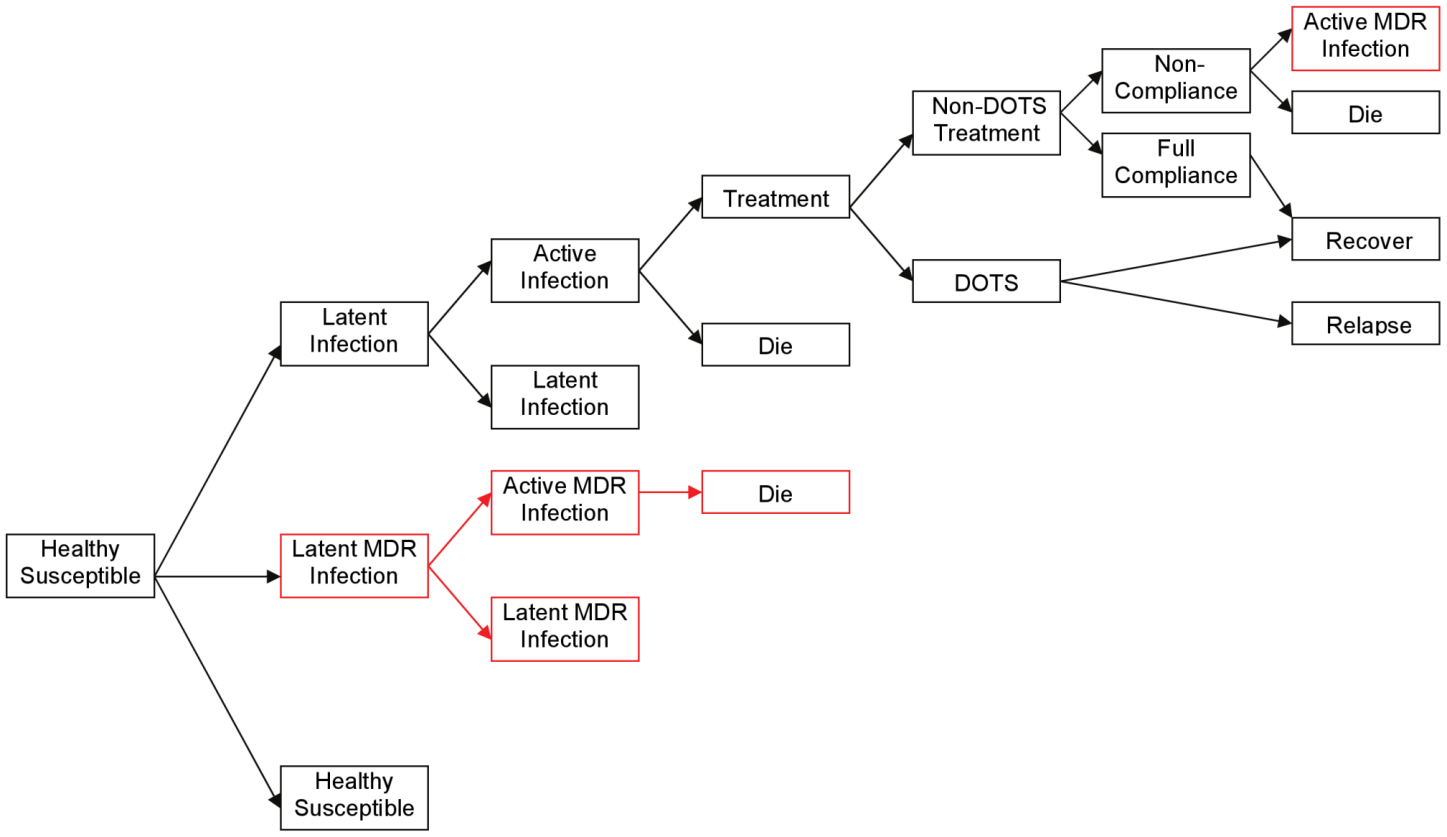
How agents progress in the model. While different in mechanics, the model has states most similar to the Cohen and Murray model. Healthy agents come into contact with actively infected agents (either drug-susceptible or MDR) and can either be infected and develop latent TB or remain healthy. Latently infected agents suffer a 10% per year chance of developing active TB for the first 10 years and a 5% per year chance of developing active TB after 10 years. Actively infected agents with drug-susceptible TB will either receive treatment or die. (Spontaneous cures of TB are possible but were not included for lack of space). Treated agents receive either DOTS or non-DOTS treatment. DOTS-treated agents recover and remain non-infective but have a 1% per year relapse rate. Non-DOTS, agents can be fully compliant, and act the same way as DOTS treated agents, or have user-determined degrees of non-compliance. Non-compliant agents have a probability of developing MDR-TB that reaches a maximum of 1% when agents take every other dose (50% compliance). DOTS never leads to MDR-TB because by definition every dose is observed and non-compliance is impossible. Once MDR-TB develops it has user-determined fitness cost which affects the probability of its spread to a susceptible host and its progression from latent to active.

The population is kept constant at 1000 people throughout time. Each person is modeled individually. Each individual's TB status is updated 10 times per year. At each clock interval each individual obtains random draws from probability distributions governing contact between susceptible and infectious individuals and governing the odds that infected individuals will have progressive disease. All state transitions are calculated one individual at a time, ten times per year for 500 years. The boxes in Figure 1 display the various TB states that are tracked for each individual and the arrows depict the stochastically determined transition probabilities. At the start of each run of the model it is assumed that 50% are latently infected with TB and the other 50% are uninfected and susceptible to TB (See Figure 1). This high prevalence of latent TB was observed in many real populations prior to widespread antimicrobial treatment [12], [13].

For the first 10 years of latent TB, patients experience a 1% annual rate of progressing to active TB and 0.5% thereafter [14]. From active TB, agents can die or potentially be detected by a TB program and then started on treatment. Before receiving treatment or dying, active TB patients can cause latent infections in susceptible patients at a rate of 1.3 secondary cases per index case.

MDR-TB spreads the same way as drug-susceptible TB, however, MDR-TB can have a fitness cost which reduces both its infectivity and the probability it will progress out of latency. Another obstacle is the immunity provided by having drug-susceptible TB. When the protective effect of drug-susceptible TB is set at 100%, any contact between an active-MDR case and a drug-susceptible case never results in MDR infections. In other sets of runs, the protective effect is set to other values (75% and 50%) so that contact between active-MDR cases and drug-susceptible cases can result in MDR-infection albeit at a reduced rate.

All agent-to-agent disease transmission is governed by probability distributions calibrated to reproduce known TB transmission rates. Parameters fall into two general categories: biological factors that are uncontrollable from a policy maker's view and treatment factors, amenable to policy. Biological factors include infectivity, mortality, activation of drug-susceptible TB, and probability of a new strain of MDR-TB to arise based on non-compliance. Treatment factors include case detection and compliance. (See Table 1) Case detection is defined as correct determination that a diseased individual has active TB. Compliance is defined as the proportion of prescribed drugs ingested by the patient from 0 percent to 100 percent. The states and pathways listed in Figure 1 are the same states used in prior models of MDR-TB transmission [15], [16], [17] The model differs from the Dye model due to the absence of a separate latent state characterized by rapid breakdown [18]. The model differs from Cohen/Murray because only one MDR strain is modeled at a time [17].

**Table 1:** Variables, settings, and sources of parameters in the model.

Treatment Variables	Base Case	Range Tested	Source
Probability TB case receives any treatment	50%	25–75%	
Probability treated agent receives DOTS	75%	50–100%	
The percent of prescribed doses taken by non-compliant agents receiving non-DOTS treatment	50%	25–75%	
**Biological Variables**			
Probability of latent infection in susceptible agent after contact with active drug-susceptible TB	1.3 secondary cases per primary case of TB 1		Blower & Chou, 2004 [36]
Case Fatality of Untreated Active Drug-susceptible TB	50% per year		Murray, Styblo, & Rouillon, 1990 [37]
Probability of progression from latent drug-susceptible TB to active TB	1% per year for first 10 years		Styblo, 1991 [14]
	0.5% per year after 10 years		
**MDR-Variables**			
Relative Fitness 2	75%	50–100%	
Immunogenicity multiplier for the probability that a latent drug-susceptible case can be infected by an MDR case 3	75%	50–100%	

1 This infectivity is calibrated such that one index case creates on average 1.3 secondary cases in a population of 1000 healthy susceptibles and conforms to Blower (2004) [36].

2 Fitness is a multiplier that determines both the probability of transmission to susceptibles and progression from latent to active in MDR cases.

3 DNA fingerprinting of active TB cases shows that it is rare that patients are infected with multiple strains simultaneously [22] making it plausible that latent infection offers immune protection. Evaluating the potential importance of this phenomenon is one of the key contributions of this model.

The biological effects highlighted by the model are 1)Effects on the frequency of new MDR-strain emergence due to poor adherence to drug treatment by TB patients; 2)Effects on the extent of spread of new strains due to a potential protective benefit from immunity due to prior latent infection with TB; and 3)Effects on the extent of spread of new strains attributable to hypothesized reduction in virulence that mycobacteria sustain in order to maintain their resistance to antimicrobials, this reduction is known as a “fitness cost”. The importance of these three elements is discussed below.

The simulation is programmed in NetLogo software [19]. The full model has been posted in the public domain at mdr.tbtools.org. Agent based models in NetLogo depict independent individuals interacting with their neighbors in space and time following simple rules that the individual agents follow probabilistically. The model follows the TB status of every individual over time from birth to death. New individuals enter and exit the model, and each generation's epidemiological environment reflects the TB prevalence established by the contingent history of prior generations. This allows the realistic depiction of multi-century epidemics like TB. The inherent random variation that occurs across populations and over time makes each run of the model different. Sensitivity analysis is based on running multiple iterations with systematically differing parameter settings.

### The effect of poor compliance

Directly observed therapy short course (DOTS) is one way to prevent non-compliance. Ideally, DOTS therapy, being directly observed, ensures that a patient takes all of their doses, completely eliminating any non-compliance whatsoever. [20] Non-compliant agents have a probability of developing MDR-TB based on the degree of non-compliance. Probability for a new strain of MDR-TB to arise in any given case is set at a maximum when compliance implies that agents took only half of their antibiotics. If there is 0% compliance then the bacteria infecting the agent has no pressure to develop resistance. If there is 100% compliance then this scenario can be assumed to be exactly like ideal DOTS.

Cases that are not directly observed may be less compliant, or they may visit clinicians who use mono-therapy. Either way they have a higher chance to later develop MDR-TB [21]. DOTS treated agents have a reduced mortality and have a small probability of relapse as shown in Table 1.

### Protective benefits of drug-susceptible infections with drug-susceptible TB

It is widely believed that having drug-susceptible infection from one unique strain of mycobacterium tuberculosis (MTB) grants an individual full or partial immunity against other different strains of MTB. Infection with multiple strains is rare [22] although exogenous re-infection has been documented [23], [24], [25] Understanding the protective benefits of drug-susceptible infection is important because the prevalence of drug-susceptible MTB is at a historical low in high-income countries. As the prevalence of drug-susceptible MTB declines, vulnerability to an imported MDR-strain may escalate.

### Fitness costs

In order for tuberculosis to develop drug resistance in any form, it must undergo genetic changes [20]. The term. “*fitness cost*” describes a situation where the genetic changes granting drug resistance also reduce the ability of MTB to reproduce within its host population. For example, isoniazid relies on the catalase-peroxidase activity controlled by the *katG* gene to transform the drug from inactive to active form. Typically an isoniazid-resistant strain will have some gene insertion or deletion that reduces its *katG* functionality [17]. Human and animal studies on isoniazid-resistant strains have demonstrated some, but not all, strains to be less pathogenic than drug-susceptible (wild-type) tuberculosis [20], [26], [27]. Guinea pigs infected with highly resistant strains had a 100% survival rate, while guinea pigs infected with moderately resistant strains typically died after 33–43 days [20]. Guinea pigs infected with drug-susceptible TB typically died after 12–19 days [20].

### Data collation

Table 1 shows the model's parameters with ranges and sources. The parameters were applied to the 1000 agents according to the probability distributions shown in the table. During each run of the model data were collected on the number of agents in each category at each time. The outcomes of interest are the percent of total agent years spent in MDR-categories, the number of runs where MDR prevalence exceeded 5% (these are counted as having created an MDR-TB epidemic), and the number of new developments (index cases) of MDR-TB.

### Scenarios and definitions

Nine policy regimes were constructed as three levels of case detection stratified by three levels of non-compliance to treatment. Case-detection was set so that 25%, 50% or 75% of all agents with active tuberculosis received treatment. The proportion of non-compliant cases was set so that 25%, 50%, or 75% of agents within a policy scenario were non-compliant to their drug regimen.

Each of the nine policy regimes was run in each of nine different biological regimes for a total of 81 separate scenarios. The biological regimes were depicted by three different fitness settings stratified by three different levels of immunogenic TB infection. MDR-TB could be 100% as fit, 75% as fit, or 50% as fit as drug-susceptible strains. Drug-susceptible infection could confer 50%, 75%, or 100% immunity from MDR-TB infection. With 50% immunity a susceptible individual who contacted an active case of TB would have half the probability of becoming infected compared to an individual with no immunity. Nine policy regimes times nine biological regimes produced 81 permutations of the model. Each permutation was run 100 times and the results summarized. The use of 100 iterations enabled the bootstrap computation of confidence intervals based on the standard deviation of observations across the iterations of each scenario.

## Results

**Figure 2:**
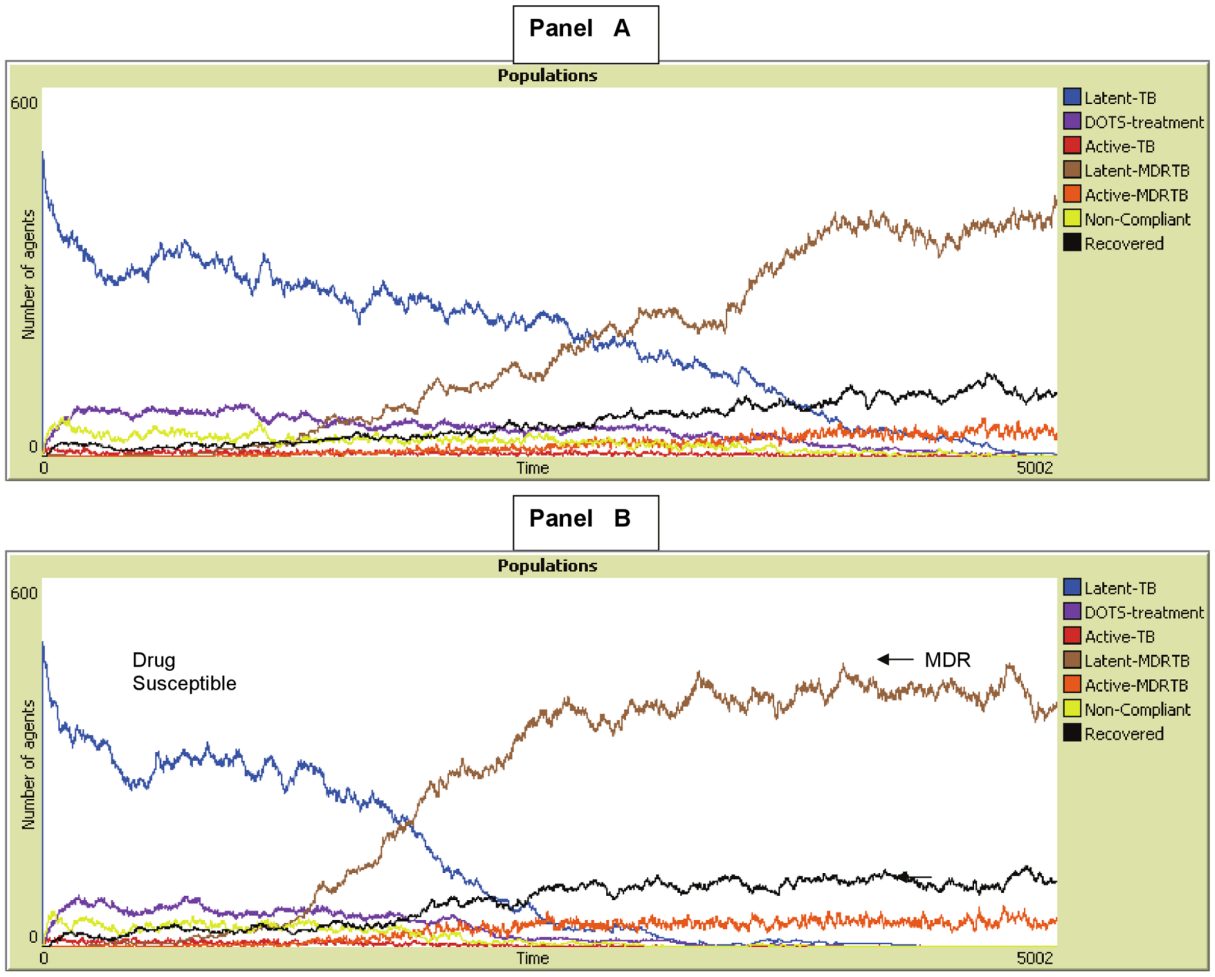
Treatment Program with 75% non-compliance and 75% case-detection 100% fitness. These panels are only 2 unique examples out of 200 runs. In panel A, drug-susceptible is 100% immunogenic, while in panel B it is only 75% immunogenic. Note that with less immunogenic drug-susceptible TB, MDR-TB takes over more rapidly, and with higher prevalence of drug-susceptible TB present.

**Figure 3:**
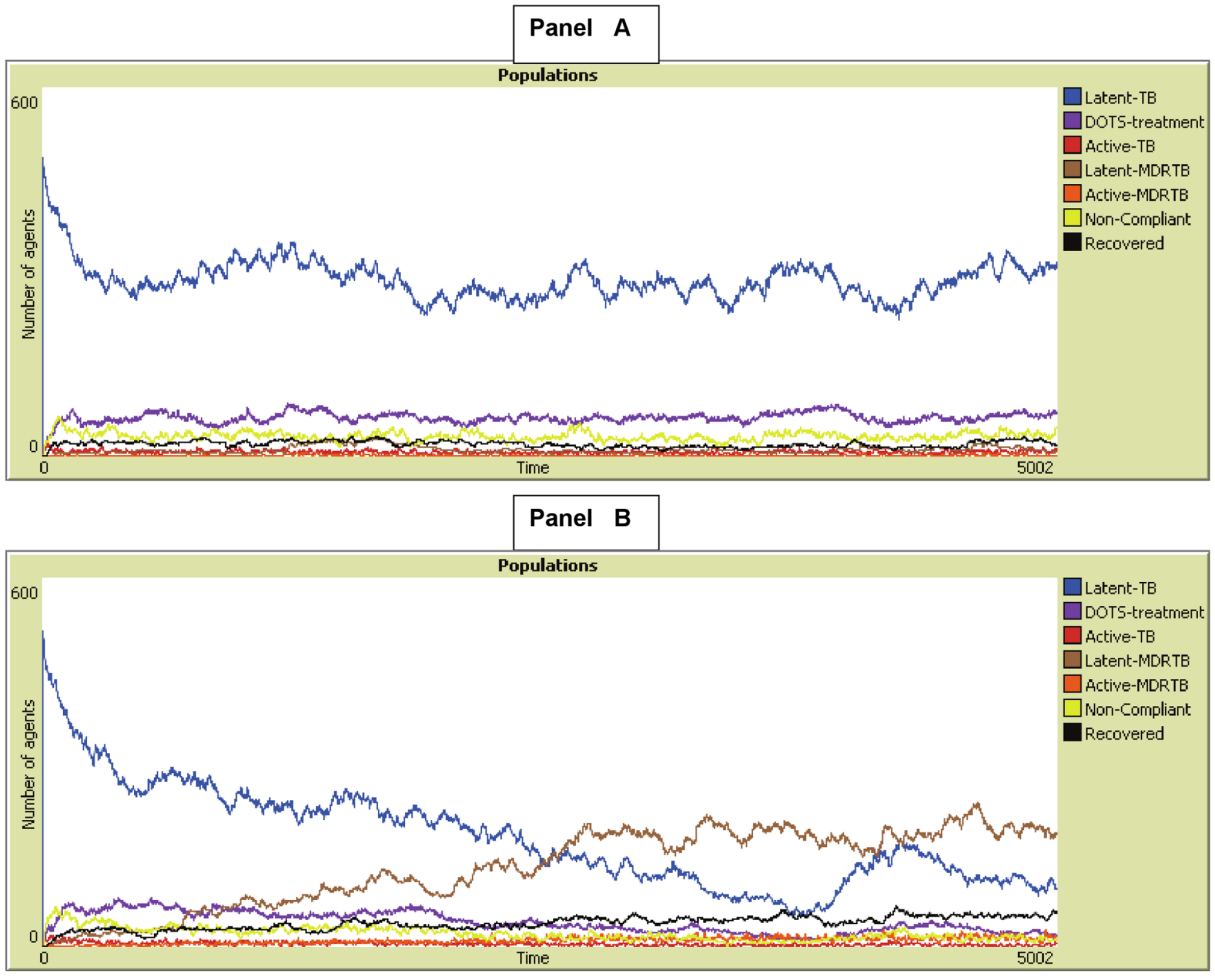
Treatment Program with 75% non-compliance and 75% case-detection and MDR with 75% of the fitness of drug susceptible TB. In panel A, drug-susceptible TB is 100% immunogenic, while in panel B it is only 50% immunogenic. In the A panel, MDR-TB is not fit enough to cause an epidemic given that drug-susceptible prevalence is high, however with a reduction in immunogenicity, drug-susceptible TB does not produce herd-immunity. This allows the less fit strain of MDR-TB to cause an epidemic.

Figures 2 and 3 show some typical graphs of MDR-TB epidemics that occurred under the various treatment programs illustrating general trends in MDR-epidemics. These graphs illustrate a *window of vulnerability* for MDR-TB where there must be enough drug-susceptible TB to create MDR-TB index cases, but not too much drug-susceptible TB to generate host immunity that inhibits the spread of MDR-TB. With less immunogenic drug-susceptible TB, higher prevalence is needed to inhibit the spread of MDR-TB strains after they emerge.

**Figure 4:**
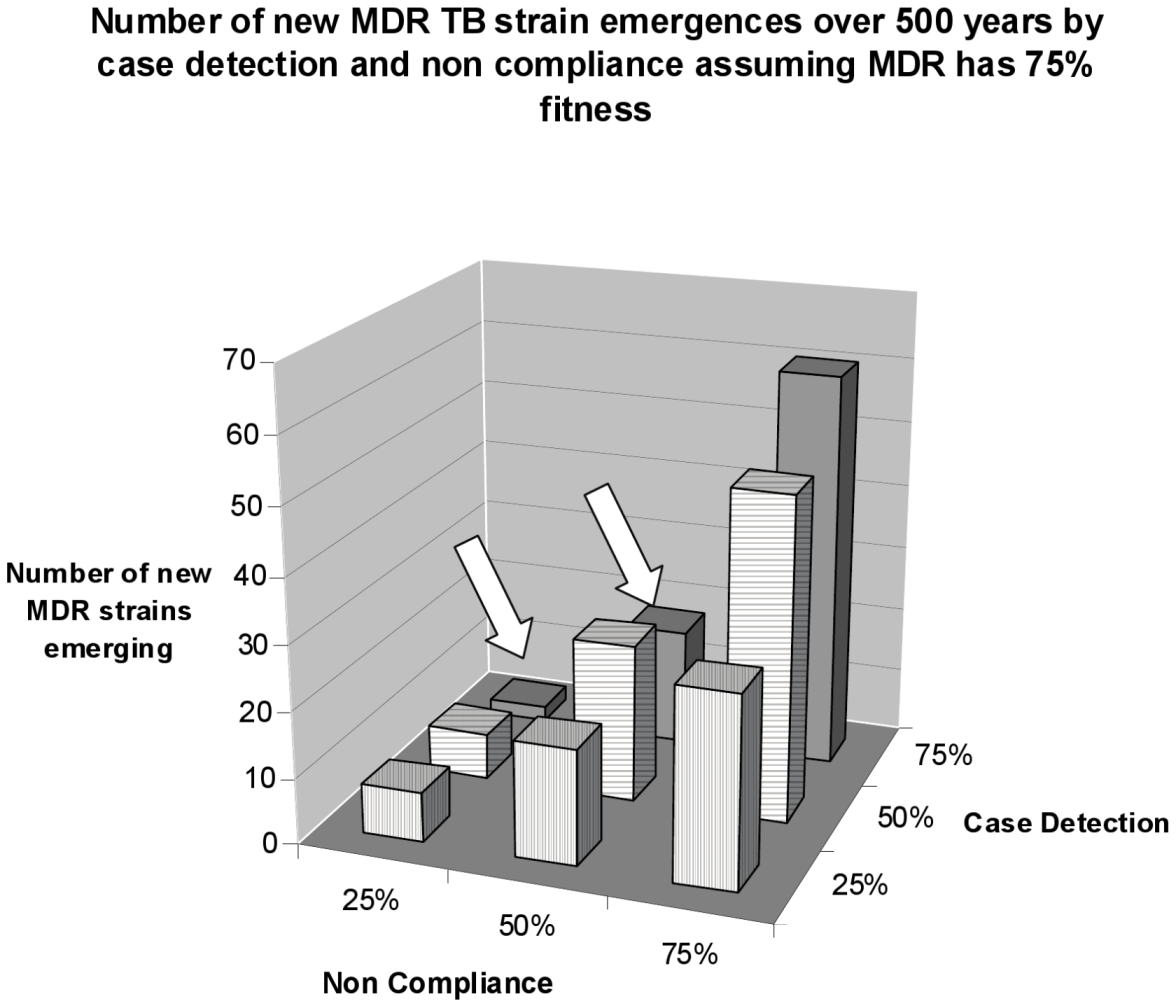
New MDR strain emergence by rates of non-compliance and case detection. Within each case-detection level, worse non-compliance always leads to more new MDR strains emerging. This implies that when non compliance is high (i.e. at 75% non-compliance) detecting more cases leads to more MDR emergence. As noted by arrows, with non-compliance down to 50% or 25% better case detection no longer leads automatically to more emerging strains of MDR TB. The reason is that there are fewer drug susceptible TB cases for MDR-TB to emerge from when non-compliance is low and case detection is high.

Figure 4 shows in three dimensions, the independent effects of compliance and case-detection on the probability of an MDR-TB epidemic. Low case detection rates allow drug-susceptible TB to flourish. But when TB cases are not detected they are not treated and MDR-TB cannot emerge unless cases get treated. The model shows that with low rates of case detection, the resulting high prevalence of drug-susceptible TB can prevent the rampant spread of MDR-TB, depending on its immunogenicity. If one examines the rows of bars in Figure 4 from front row to back row the slope across the rows becomes steeper in the back row (where case detection is 75%) compared to the front row where case detection is 25%. The steepness is an indicator of how important compliance is in preventing MDR-TB epidemics and our results show that compliance is more important when case detection rates are high.

The effects of increasing case-detection are non-linear when more agents do not comply with therapy. When non-compliance rates are 50%, intermediate case-detection rates lead to more emergent strains of MDR-TB than when there is lower or higher case-detection. This paradox is discussed below.

**Figure 5:**
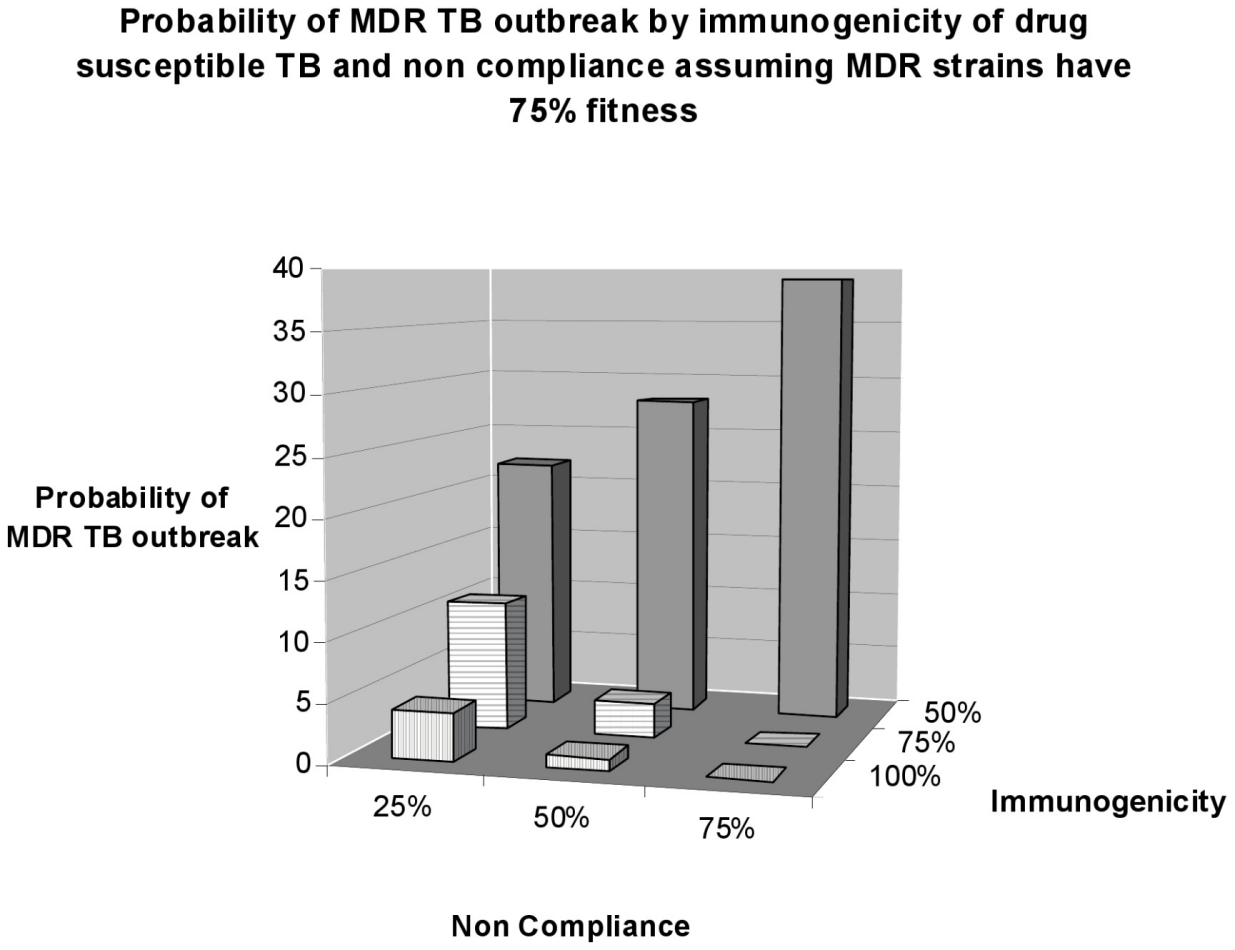
Probability of MDR-TB outbreaks by non-compliance and the degree of immunogenicity of drug-sensitive TB infection. Probability of an MDR TB outbreak is highest when patients have highest rates of non-compliance (e.g. 75% non-compliant) and when immunogenicity of prior infection with TB is low (e.g. 50% of patients are protected from successive infections by prior infections.) The back row of the graph shows that when with low immunogenicity there is a systematic rise in probability of MDR-TB outbreaks as rates of non-compliance rise. As shown in the front two rows, this relationship is reversed when immunogenicity is higher. When immunogenicity is high non-compliance serves primarily to spread the immunizing strain of drug-sensitive TB which lowers the probability of an MDR TB outbreak.

Figure 5 shows the generalized effects of immunogenicity and non-compliance on the probability of an MDR outbreak. No matter how non-compliant the population is, the less immunity afforded by drug-susceptible TB the higher the probability of an MDR outbreak. Figure 5 also demonstrates how immunogenicity parameters moderate the competition between drug-susceptible and MDR-TB for hosts. The front two rows of Figure 5 show that when drug-susceptible TB confers 75 to 100% immunity, higher rates of non-compliance create more cases of drug-susceptible TB and more individuals immune to MDR-TB, hence lowering the probability of MDR-TB outbreaks. However, as shown in the back row of Figure 5, when immunogenicity is 50%, the human ecology is such that the greater numbers of newly emergent MDR-TB strains arising, coupled with rising rates of non-compliance are able to spread throughout the population with greater frequency. The data supporting Figures 4 and 5 and a summary of all 81 policy and biological regimes is given in tables 2 and 3.

**Table 2:** Summarizes 8100 runs giving the mean number of runs leading to MDR epidemics as defined by WHO standards.

Proportions of Runs Ending in MDR-TB epidemics																								
**Immunogenicity**	0.50								0.75								1.00							
**Fitness**	0.50		0.75			1.00			0.50		0.75			1.00			0.50		0.75			1.00		
**25% Case Detection**																								
**Non- Compliance**																								
0.25	0.00	± NA	5.00	±	*0.48*	*80.00*	*±*	*1.60*	0.00	± NA	0.00	±	NA	62.00	±	*2.36*	0.00	± NA	0.00	±	NA	33.00	±	*2.21*
0.50	0.00	± NA	12.00	±	*1.06*	*98.00*	±	*0.20*	0.00	± NA	0.00	±	NA	90.00	±	*0.90*	0.00	± NA	0.00	±	NA	47.00	±	*2.49*
0.75	0.00	± NA	13.00	±	*1.13*	*100.00*	±	*NA*	0.00	± NA	0.00	±	NA	96.00	±	*0.38*	0.00	± NA	0.00	±	NA	37.00	±	*2.33*
**50% Case Detection**																								
**Non- Compliance**																								
0.25	0.00	± NA	22.00	±	*1.72*	*76.00*	±	*1.82*	0.00	± NA	11.00	±	*0.98*	82.00	±	*1.48*	0.00	± NA	4.00	±	*0.38*	72.00	±	*2.02*
0.50	0.00	± NA	28.00	±	*2.02*	*99.00*	±	*0.10*	0.00	± NA	3.00	±	*0.29*	98.00	±	*0.20*	0.00	± NA	1.00	±	*0.10*	86.00	±	*1.20*
0.75	0.00	± NA	39.00	±	*2.38*	*100.00*	±	*NA*	0.00	± NA	0.00	±	*0.00*	100.00	±	*0.00*	0.00	± NA	0.00	±	*0.00*	92.00	±	*0.74*
**75% Case Detection**																								
**Non- Compliance**																								
0.25	0.00	± NA	19.00	±	*1.54*	*48.00*	±	*2.50*	0.00	± NA	15.00	±	*1.28*	45.00	±	*2.48*	0.00	± NA	14.00	±	*1.20*	34.00	±	*2.24*
0.50	0.00	± NA	55.00	±	*2.48*	*98.00*	±	*0.20*	0.00	± NA	45.00	±	*2.48*	98.00	±	*0.20*	0.00	± NA	11.00	±	*0.98*	95.00	±	*0.48*
0.75	0.00	± NA	61.00	±	*2.38*	*100.00*	±	*NA*	0.00	± NA	8.00	±	*0.74*	100.00	±	*0.00*	0.00	± NA	0.00	±	*0.00*	98.00	±	*0.20*

The 95% confidence level for each estimate is given based on the standard deviations observed in 100 iterations in the model calculated as (p)(1-p)/n^∧^0.5. Italicized figures are representative of situations with highly prevalent HIV in which immunogenicity of TB is low and the fitness of the organism is high.

**Table 3:** Summarizes 8100 runs giving the mean number of unique MDR strain emergences.

Average Number of MDR-TB index cases per iteration																											
**Immunogenicity**	50%									75%									100%								
**Fitness**	50%			75%			100%			50%			75%			100%			50%			75%			100%		
**25% Case Detection**																											
Non- Compliance																											
25%	7.71	±	0.51	7.73	±	0.50	4.28	±	0.40	7.67	±	0.51	7.47	±	0.53	5.17	±	0.44	7.99	±	0.52	7.84	±	0.55	6.42	±	0.51
50%	17.67	±	0.77	17.04	±	0.80	7.08	±	0.61	18.02	±	0.82	17.56	±	0.78	9.92	±	0.72	18.21	±	0.87	17.81	±	0.79	15.3	±	0.88
75%	29.54	±	1.11	28.38	±	1.15	10.88	±	0.71	30.71	±	1.10	29.13	±	0.93	15.29	±	1.09	29.79	±	1.06	28.99	±	0.95	25.91	±	0.90
**50% Case Detection**																											
**Non- Compliance**																											
25%	7.37	±	0.61	6.42	±	0.52	4.17	±	0.43	7.41	±	0.56	6.99	±	0.55	4.53	±	0.39	7.28	±	0.54	7.03	±	0.56	5.16	±	0.42
50%	24.71	±	0.94	21.64	±	0.99	8.7	±	0.64	24.28	±	1.05	24.09	±	0.91	10.54	±	0.87	24.89	±	1.05	24.41	±	0.95	15.11	±	1.14
75%	51.1	±	1.40	44.56	±	1.49	14.03	±	0.94	51.14	±	1.43	49.48	±	1.40	19.94	±	1.18	50.83	±	1.47	50.18	±	1.35	36.61	±	1.59
**75% Case Detection**																											
**Non- Compliance**																											
25%	2.41	±	0.32	2.4	±	0.33	2.06	±	0.27	2.3	±	0.32	2.25	±	0.31	2.06	±	0.32	2.22	±	0.33	2.15	±	0.28	2.11	±	0.29
50%	21.03	±	1.01	17.09	±	1.04	7.88	±	0.70	21.29	±	0.93	17.93	±	1.03	8.99	±	0.73	21.94	±	1.06	19.69	±	1.01	11.36	±	0.86
75%	63.76	±	1.55	51.66	±	1.76	16.12	±	0.90	62.71	±	1.62	60.48	±	1.54	21.78	±	1.35	64.58	±	1.64	62.18	±	1.84	34.94	±	1.90

95% confidence interval shown. Note that while there may be lower numbers of index cases where MDR-TB is more fit, this is due to MDR-TB outcompeting drug-susceptible TB thus reducing the potential of index cases to develop.

Variation in the outcome of runs tended towards a normal distribution for each of the tested values. Standard deviations were low, indicating that averages attained were reliable outcomes from the model. Figures 4 and 5 show the impact of changing the parameters in the model and provide a sensitivity analysis of how important both policy and biological parameters are in supporting or inhibiting the emergence of MDR TB strains.

Under most treatment policies, lower immunogenicity of drug-susceptible TB makes MDR-epidemics more probable. However, the extent to which immunogenicity affected the probability of MDR-epidemics diminished as MDR-TB became relatively more fit. Fitness cost appeared to be the greatest obstacle to the spread of MDR-TB. With MDR-TB set to be 50% as fit as drug-susceptible TB, MDR-TB could not produce a single epidemic in the 2700 iterations studied with fitness at this low setting. Increased MDR-TB fitness almost always led to increased probability of MDR-TB epidemics. Tables 1 and 2 summarize the detailed results of 8100 runs (9 policies scenarios×9 biological sets×100 iterations). Additional information is available in Appendix S1 entitled “Appendix to Heightened vulnerability to MDR-TB epidemics after controlling drug-susceptible TB”.

## Discussion

The results of the model stress the need to maintain linkage between success in TB case detection and success in reducing non-compliance with TB treatment. As new TB diagnostics are developed in coming decades, one must consider the implications of deploying them in areas that cannot maintain high rates of compliance with treatment. Minimum standards for success in TB treatment need to accompany phasing in better diagnostics. The worst scenario in our model in terms of MDR-TB emergence is high case detection rates combined with high non-compliance rates as shown in the back row, far right of Figure 4. Although a heavy focus on compliance without case detection will not stem the general epidemic of drug susceptible TB, it will keep MDR-TB in check as shown in the bars along the left edge of Figure 4. The interesting aspect is that at 25% and 50% rates of non-compliance, MDR-strains emerge more readily when case detection rates are intermediate than when they are low or high. This occurs because intermediate rates of case detection are more likely to open the window of vulnerability where general TB prevalence is low enough to keep immunity down but there are enough non-compliant cases circulating in the community poised for breeding MDR-strains.

An exciting discovery from the model was the presence of windows of vulnerability for MDR-TB epidemics to emerge. These windows occur after the prevalence of TB infection and the immunity it confers has been reduced to less than 30% prevalence of latent TB leaving most of the population vulnerable. New emergent strains of MDR-TB could only perpetuate after this window of vulnerability opened. The window closes when countries bring their rates of prevalent TB below 5%. At these low rates of TB prevalence, there are too few cases of active TB for poor treatment to have a chance to yield MDR-strains. Imported MDR strains would be a larger threat, but these are not covered in the model.

MDR-TB epidemics are dependent on the dynamics of competition between MDR and drug-susceptible TB. (See figure 5). Both drug-susceptible and MDR-TB compete for a common resource: susceptible hosts. TB treatment policies in the model alter how well MDR-TB competes against drug-susceptible TB. Weak treatment policies that allow non-compliance, select against drug-susceptible TB and permit resistance to emerge, but they only give MDR-TB a foothold when most of the population has not had a prior infection with TB.

Paradoxically, the model shows that a TB program's success against drug-susceptible TB makes a population vulnerable to MDR-TB. A high prevalence of drug-susceptible TB can provide herd-immunity against MDR-TB, in effect preventing MDR-TB from progressing out of static low prevalence. The more immunogenic drug-susceptible TB is, the less the prevalence needed to achieve herd-immunity. Treatment that reduces the prevalence of drug-susceptible TB while the population is exposed to a low prevalence MDR-TB for decades facilitates MDR-TB epidemics by providing MDR-TB with more susceptible hosts and the time needed to perpetuate. However, allowing drug-susceptible TB to remain unchecked is not an acceptable policy strategy. Speed is the key to a treatment policy that minimizes MDR-TB development and is effective against drug-susceptible TB. Rapid elimination, however, requires both high case-detection and compliance rates. High case-detection rates coupled with high rates of non- compliance yield high rates of MDR-TB creation as shown in Figure 4. High compliance rates coupled with low case-detection are ineffective in controlling drug-susceptible TB. The key finding regarding policy is to first develop consistent and rigorous DOTS which must be maintained throughout the course of improving case detection. Lapsed DOTS while at intermediate levels of case detection is more permissive of MDR-TB, more so than at low and high levels of i case detection. With low case detection the high levels of immunity from latent drug-susceptible TB hold MDR-TB in check. With high case detection there is less protection from latent immunity.

In this model as well as other models of MDR-TB, biological fitness costs can be the greatest natural obstacle to the development of MDR-TB epidemics. If MDR-TB strains are substantially unfit then even under less vigilant policy regimes they cannot spread. In our model, a 25% reduction in fitness (from 100% to 75%) with 50% case-detection and 50% non-compliance lowers probability of MDR-TB an epidemic from 98% to 3%. It remains an important goal to quantify the relative fitness of MDR-TB strains. However, our model highlights that as the quality of treatment programs improves, the importance of all of the biological factors of TB diminishes. Good treatment means there are fewer organisms that can develop resistance and fewer cases of bad treatment to provide an opportunity for resistance to emerge.

### Strengths and limitations

The model is limited in that innate differences in host immunity (e.g. due to HIV/AIDS) are ignored. Host immunity plays a major role in the development and spread of tuberculosis as is well known from the spread of MDR-TB from immunocompetent to HIV infected patients during an outbreak in New York [28]. Likely, the fitness cost of drug resistance would be moderately negated, and the immunogenic effect of latent TB would be lessened in HIV patients. In regards to this, the simulations where drug susceptible strains are less immunogenic and where MDR strains are more fit would be informative about how the presence of prevalent HIV could affect the model. The inclusion of HIV in subsequent models therefore may reduce the protective effect of drug susceptible TB on a population level and increase the spread of less fit strains of MDR-TB. On a population level this reduces the protective effect of latent drug susceptible TB infection in regions with highly prevalent HIV infection. HIV co-infection can be added to later versions of the model, but simplicity was intentionally stressed in this initial version.

In addition, the model makes no provision for second-line drugs to treat MDR-TB or for the introduction of new drugs active against both drug-susceptible and MDR-TB [29], [30], [31], [32]. In effect the modeled version of MDR-TB is essentially extensively-drug resistant (XDR) TB (resistant to at least isoniazid, rifampicin as well as any quinolone and at least one injectable second line agent). In addition, each new MDR-TB strain has the same virulence. Whereas in reality, independent cases of MDR-TB would be likely to have their own unique antibiotic susceptibility as well as fitness costs [17]. It is reasonable to assume that over time, independently arising strains that perpetuate would show convergent evolution towards similar compensatory mutations, however this is likely to be a very slow process.

Another central assumption of the model is that the net death rate is equivalent to the net birth rate, so population remains constant. Population density affects the daily contact rate of agents and therefore the number of secondary cases developed per index case, and by setting population and therefore population density as a constant, the model removed a dynamic part of the epidemiological progression [33], [34].

Another important limitation is that there is no migration because the focus is on the conditions for MDR-strains to emerge and spread within a single population. Focusing on a population's resistance to immigrant strains of MDR-TB would be a straightforward extension, but much is learned from the current model by studying the ability of native MDR-strains to arise and perpetuate. The principal findings regarding how TB treatment policies affect the probability of new MDR strains arising would not be changed by the inclusion of population growth and migration. The prevalence of drug susceptible strains, regardless of population dynamics, determines the protective benefit from latent TB. This study concludes that when current knowledge about TB epidemiology is condensed into a computer model it reveals that MDR strains are most likely to emerge and spread in populations that have just begun to enjoy successful control of drug susceptible TB. The window of vulnerability to the spread of native MDR strains (and by extension to imported strains) is highest when few members of the population have ever been infected by TB. This underscores the current vulnerability of populations in Europe, Japan, and North America. In the 1990s the re-emergence of TB in the US was attributed to an abandonment of public health measures to control TB [35]. Paradoxically it is our current success in controlling drug-susceptible TB that makes the US population more vulnerable to both native and introduced outbreaks of MDR-TB. The most successful approach to reduce this risk would be to ensure that populations around the world combine high rates of case finding that is tightly coupled to high compliance with DOTS.

## Supporting Information

Appendix S1 Appendix to heightened vulnerability to MDR-TB epidemics after controlling drug-susceptible TB.
(0.17 MB DOC)
