# Supplementary material for: Heightened Vulnerability to MDR-TB Epidemics after Controlling Drug-Susceptible TB
Source: PLoS One. 2010 Sep 22;5(9):e12843. doi: 10.1371/journal.pone.0012843 (PMC2943899; doi:10.1371/journal.pone.0012843)
Supplement: Appendix S1 — Appendix to heightened vulnerability to MDR-TB epidemics after controlling drug-susceptible TB. (0.17 MB DOC) [file pone.0012843.s001.doc]

**Appendix to**

**Heightened vulnerability to MDR-TB epidemics after**

**controlling drug-susceptible TB**

This appendix gives summary statistics for 81 scenarios. Each one was examined with 100 iterations of the simulation yielding 8100 total. Table A1 shows the proportion out of 100 for each scenario that resulted in an MDR-TB epidemic. Table A2 shows the mean number and (SD) of new strains of MDR TB that emerged in each scenario.

Table A1 The proportion of cases (out of 100) that resulted in MDR-TB epidemics.

| **Proportions of Runs Ending in MDR-TB epidemics** | | | | | | | | | | | | | | | | | | | | |
| --- | --- | --- | --- | --- | --- | --- | --- | --- | --- | --- | --- | --- | --- | --- | --- | --- | --- | --- | --- | --- |
| **Immunogenicity** | 50% | | | | | |  | 75% | | | | | |  | 100% | | | | | |
| **Fitness** | 50% | | 75% | | 100% | |  | 50% | | 75% | | 100% | |  | 50% | | 75% | | 100% | |
| **25% Case Detection** | | | | | | | | | | | | | | | | | | | | |
| **Non- Compliance** |  |  |  |  |  |  |  |  |  |  |  |  |  |  |  |  |  |  |  |  |
| 25% | 0 | (0.00) | 5 | (2.18) | 80 | (4.00) |  | 0 | (0.00) | 0 | (0.00) | 62 | (4.85) |  | 0 | (0.00) | 0 | (0.00) | 33 | (4.70) |
| 50% | 0 | (0.00) | 12 | (3.25) | 98 | (1.40) |  | 0 | (0.00) | 0 | (0.00) | 90 | (3.00) |  | 0 | (0.00) | 0 | (0.00) | 47 | (4.99) |
| 75% | 0 | (0.00) | 13 | (3.36) | 100 | (0.00) |  | 0 | (0.00) | 0 | (0.00) | 96 | (1.96) |  | 0 | (0.00) | 0 | (0.00) | 37 | (4.83) |
|  |  |  |  |  |  |  |  |  |  |  |  |  |  |  |  |  |  |  |  |  |
| **50% Case Detection** | | | | | | | | | | | | | | | | | | | | |
| **Non- Compliance** |  |  |  |  |  |  |  |  |  |  |  |  |  |  |  |  |  |  |  |  |
| 25% | 0 | (0.00) | 22 | (4.14) | 76 | (4.27) |  | 0 | (0.00) | 11 | (3.13) | 82 | (3.84) |  | 0 | (0.00) | 4 | (1.96) | 72 | (4.49) |
| 50% | 0 | (0.00) | 28 | (4.49) | 99 | (0.99) |  | 0 | (0.00) | 3 | (1.71) | 98 | (1.40) |  | 0 | (0.00) | 1 | (0.99) | 86 | (3.47) |
| 75% | 0 | (0.00) | 39 | (4.88) | 100 | (0.00) |  | 0 | (0.00) | 0 | (0.00) | 100 | (0.00) |  | 0 | (0.00) | 0 | (0.00) | 92 | (2.71) |
|  |  |  |  |  |  |  |  |  |  |  |  |  |  |  |  |  |  |  |  |  |
| **75% Case Detection** | | | | | | | | | | | | | | | | | | | | |
| **Non- Compliance** |  |  |  |  |  |  |  |  |  |  |  |  |  |  |  |  |  |  |  |  |
| 25% | 0 | (0.00) | 19 | (3.92) | 48 | (5.00) |  | 0 | (0.00) | 15 | (3.57) | 45 | (4.97) |  | 0 | (0.00) | 14 | (3.47) | 34 | (4.74) |
| 50% | 0 | (0.00) | 55 | (4.97) | 98 | (1.40) |  | 0 | (0.00) | 45 | (4.97) | 98 | (1.40) |  | 0 | (0.00) | 11 | (3.13) | 95 | (2.18) |
| 75% | 0 | (0.00) | 61 | (4.88) | 100 | (0.00) |  | 0 | (0.00) | 8 | (2.71) | 100 | (0.00) |  | 0 | (0.00) | 0 | (0.00) | 98 | (1.40) |

Table A2 displays the average number of index cases of MDR-TB per iteration for each treatment strategy

| **Average Number of MDR-TB index cases per iteration** | | | | | | | | | | | | | | | | | | | | |
| --- | --- | --- | --- | --- | --- | --- | --- | --- | --- | --- | --- | --- | --- | --- | --- | --- | --- | --- | --- | --- |
| **Immunogenicity** | 50% | | | | | |  | 75% | | | | | |  | 100% | | | | | |
| **Fitness** | 50% | | 75% | | 100% | |  | 50% | | 75% | | 100% | |  | 50% | | 75% | | 100% | |
| **25% Case Detection** | | | | | | | | | | | | | | | | | | | | |
| Non- Compliance |  |  |  |  |  |  |  |  |  |  |  |  |  |  |  |  |  |  |  |  |
| 25% | 7.71 | (2.60) | 7.73 | (2.55) | 4.28 | (2.03) |  | 7.67 | (2.60) | 7.47 | (2.72) | 5.17 | (2.27) |  | 7.99 | (2.67) | 7.84 | (2.80) | 6.42 | (2.58) |
| 50% | 17.67 | (3.93) | 17.04 | (4.06) | 7.08 | (3.10) |  | 18.02 | (4.19) | 17.56 | (3.96) | 9.92 | (3.68) |  | 18.21 | (4.45) | 17.81 | (4.03) | 15.3 | (4.49) |
| 75% | 29.54 | (5.67) | 28.38 | (5.89) | 10.88 | (3.63) |  | 30.71 | (5.60) | 29.13 | (4.72) | 15.29 | (5.54) |  | 29.79 | (5.43) | 28.99 | (4.86) | 25.91 | (4.61) |
|  |  |  |  |  |  |  |  |  |  |  |  |  |  |  |  |  |  |  |  |  |
| **50% Case Detection** | | | | | | | | | | | | | | | | | | | | |
| **Non- Compliance** |  |  |  |  |  |  |  |  |  |  |  |  |  |  |  |  |  |  |  |  |
| 25% | 7.37 | (3.10) | 6.42 | (2.64) | 4.17 | (2.17) |  | 7.41 | (2.84) | 6.99 | (2.80) | 4.53 | (2.01) |  | 7.28 | (2.75) | 7.03 | (2.88) | 5.16 | (2.14) |
| 50% | 24.71 | (4.81) | 21.64 | (5.03) | 8.7 | (3.29) |  | 24.28 | (5.38) | 24.09 | (4.64) | 10.54 | (4.42) |  | 24.89 | (5.35) | 24.41 | (4.83) | 15.11 | (5.80) |
| 75% | 51.1 | (7.13) | 44.56 | (7.58) | 14.03 | (4.79) |  | 51.14 | (7.31) | 49.48 | (7.14) | 19.94 | (6.04) |  | 50.83 | (7.48) | 50.18 | (6.91) | 36.61 | (8.09) |
|  |  |  |  |  |  |  |  |  |  |  |  |  |  |  |  |  |  |  |  |  |
| **75% Case Detection** | | | | | | | | | | | | | | | | | | | | |
| **Non- Compliance** |  |  |  |  |  |  |  |  |  |  |  |  |  |  |  |  |  |  |  |  |
| 25% | 2.41 | (1.65) | 2.4 | (1.67) | 2.06 | (1.39) |  | 2.3 | (1.63) | 2.25 | (1.59) | 2.06 | (1.61) |  | 2.22 | (1.67) | 2.15 | (1.42) | 2.11 | (1.48) |
| 50% | 21.03 | (5.13) | 17.09 | (5.32) | 7.88 | (3.57) |  | 21.29 | (4.73) | 17.93 | (5.26) | 8.99 | (3.73) |  | 21.94 | (5.41) | 19.69 | (5.15) | 11.36 | (4.38) |
| 75% | 63.76 | (7.90) | 51.66 | (8.97) | 16.12 | (4.60) |  | 62.71 | (8.26) | 60.48 | (7.86) | 21.78 | (6.87) |  | 64.58 | (8.38) | 62.18 | (9.40) | 34.94 | (9.67) |
